# Supplementary figures and images for: Immune-Targeted Therapy with or without Transarterial Chemoembolization (TACE) for Advanced Hepatocellular Carcinoma with Portal Vein Tumor Thrombosis (PVTT): A Multicenter Retrospective Study
Source: Biomedicines. 2024 Sep 19;12(9):2124. doi: 10.3390/biomedicines12092124 (PMC11429150; doi:10.3390/biomedicines12092124)

PVTT    VP2    VP3    VP4

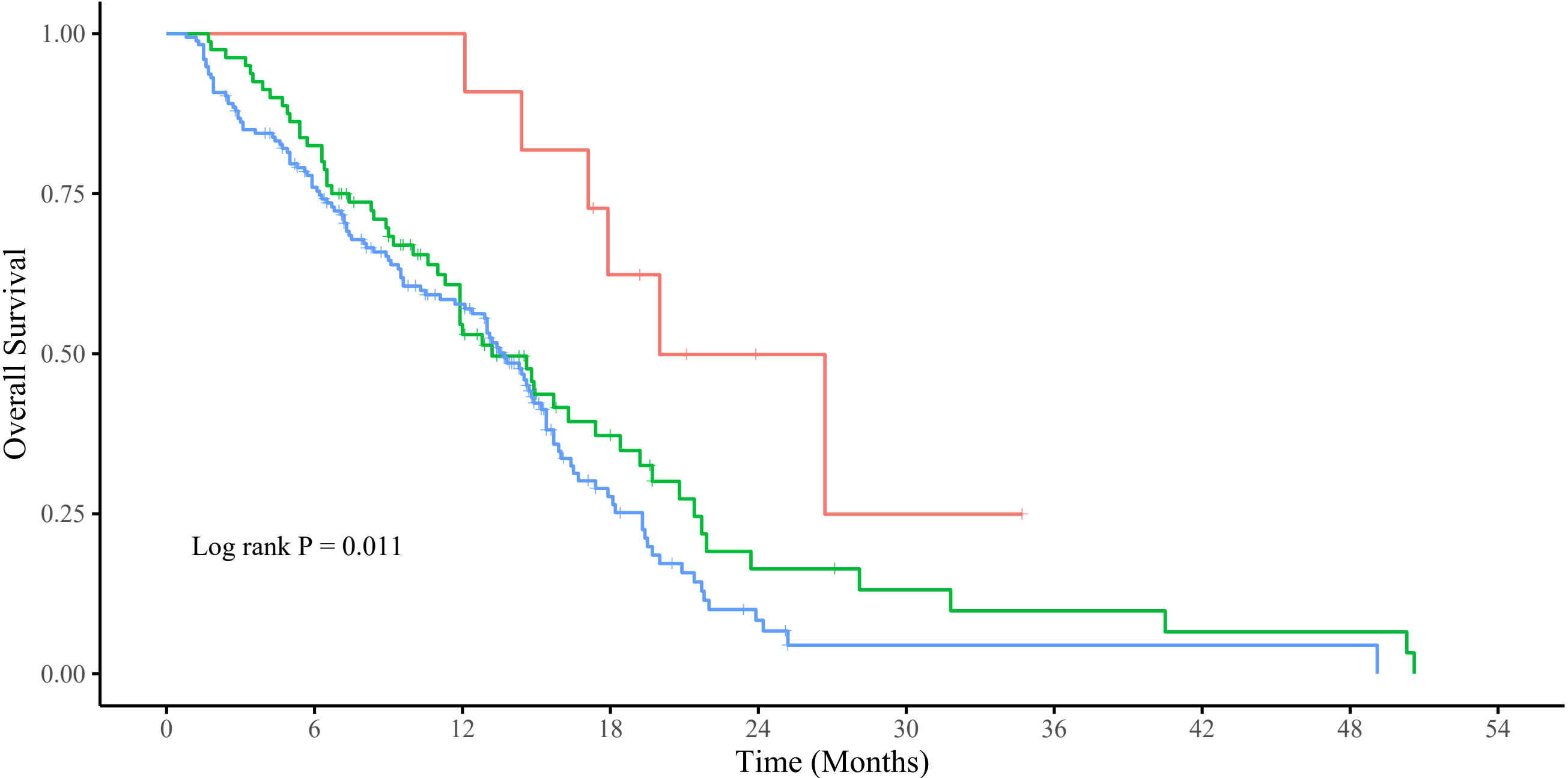

Number at risk

|     |     |     |    |    |   |   |   |   |   |   |
|-----|-----|-----|----|----|---|---|---|---|---|---|
| VP2 | 11  | 11  | 11 | 6  | 2 | 1 | 0 | 0 | 0 | 0 |
| VP3 | 80  | 66  | 35 | 17 | 6 | 4 | 3 | 2 | 2 | 0 |
| VP4 | 174 | 124 | 79 | 22 | 5 | 1 | 1 | 1 | 1 | 0 |

Supplement: Supplementary file 1 [file biomedicines-12-02124-s001.zip › Supplementary Figure S1A.pdf]

PVTT + VP2 + VP3 + VP4

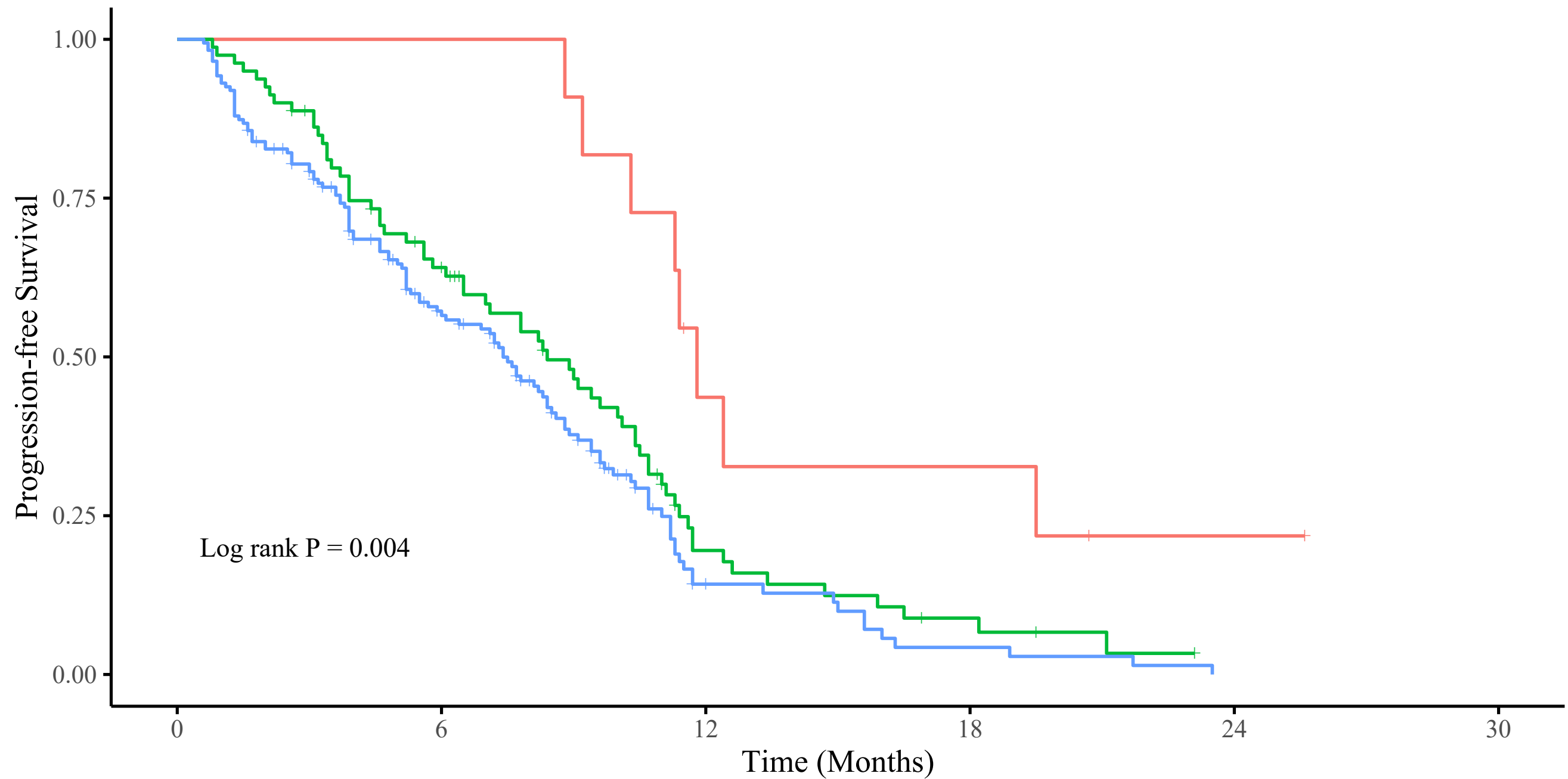

Number at risk

|     |     |    |    |   |   |   |
|-----|-----|----|----|---|---|---|
| VP2 | 11  | 11 | 4  | 3 | 1 | 0 |
| VP3 | 80  | 48 | 11 | 4 | 0 | 0 |
| VP4 | 174 | 82 | 11 | 3 | 0 | 0 |

Supplement: Supplementary file 1 [file biomedicines-12-02124-s001.zip › Supplementary Figure S1B.pdf]

PVTT + VP2 + VP3 + VP4

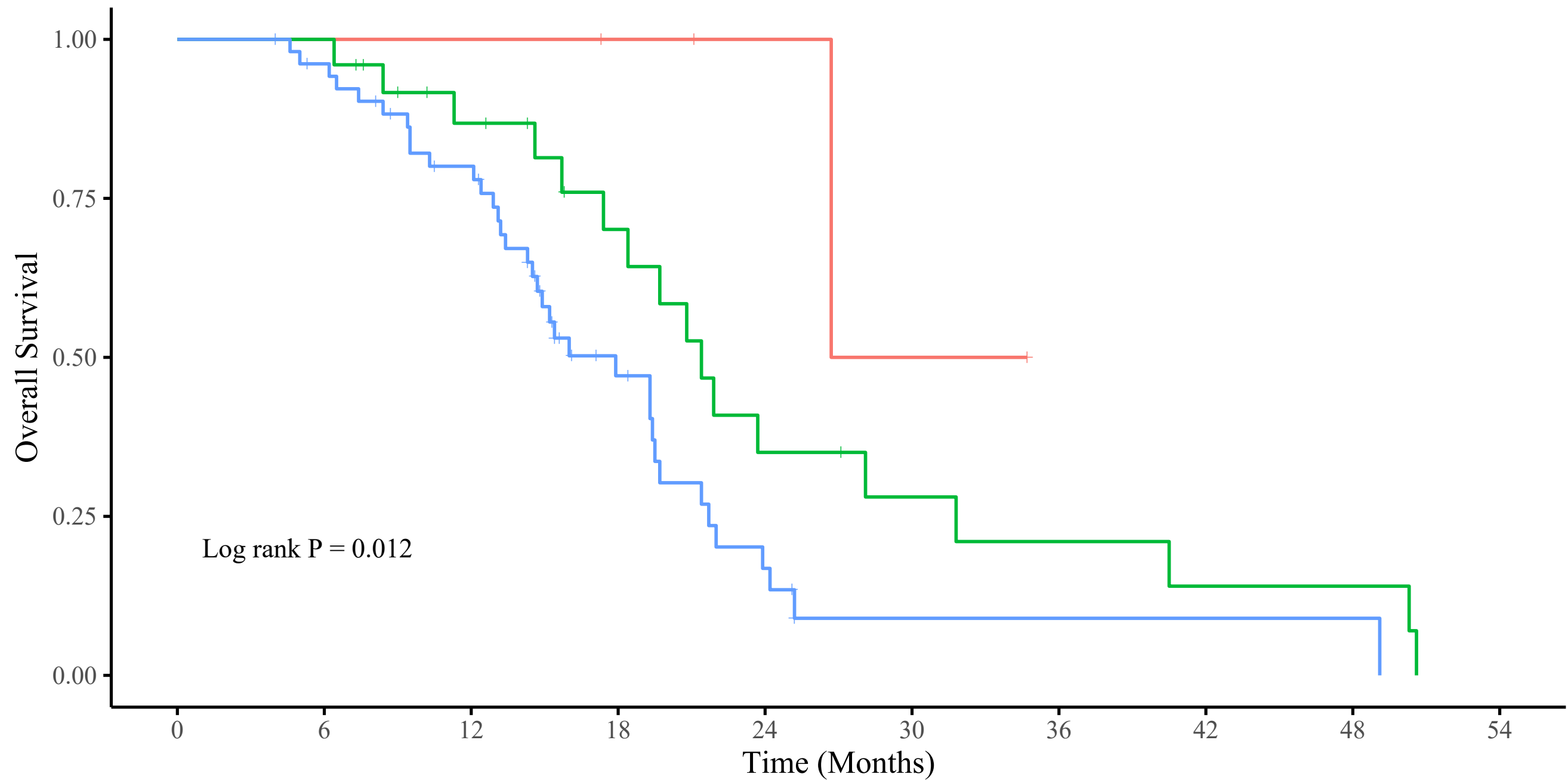

Number at risk

|     |    |    |    |    |   |   |   |   |   |   |
|-----|----|----|----|----|---|---|---|---|---|---|
| VP2 | 4  | 4  | 4  | 3  | 2 | 1 | 0 | 0 | 0 | 0 |
| VP3 | 25 | 25 | 18 | 12 | 6 | 4 | 3 | 2 | 2 | 0 |
| VP4 | 53 | 49 | 38 | 15 | 5 | 1 | 1 | 1 | 1 | 0 |

Supplement: Supplementary file 1 [file biomedicines-12-02124-s001.zip › Supplementary Figure S2A.pdf]

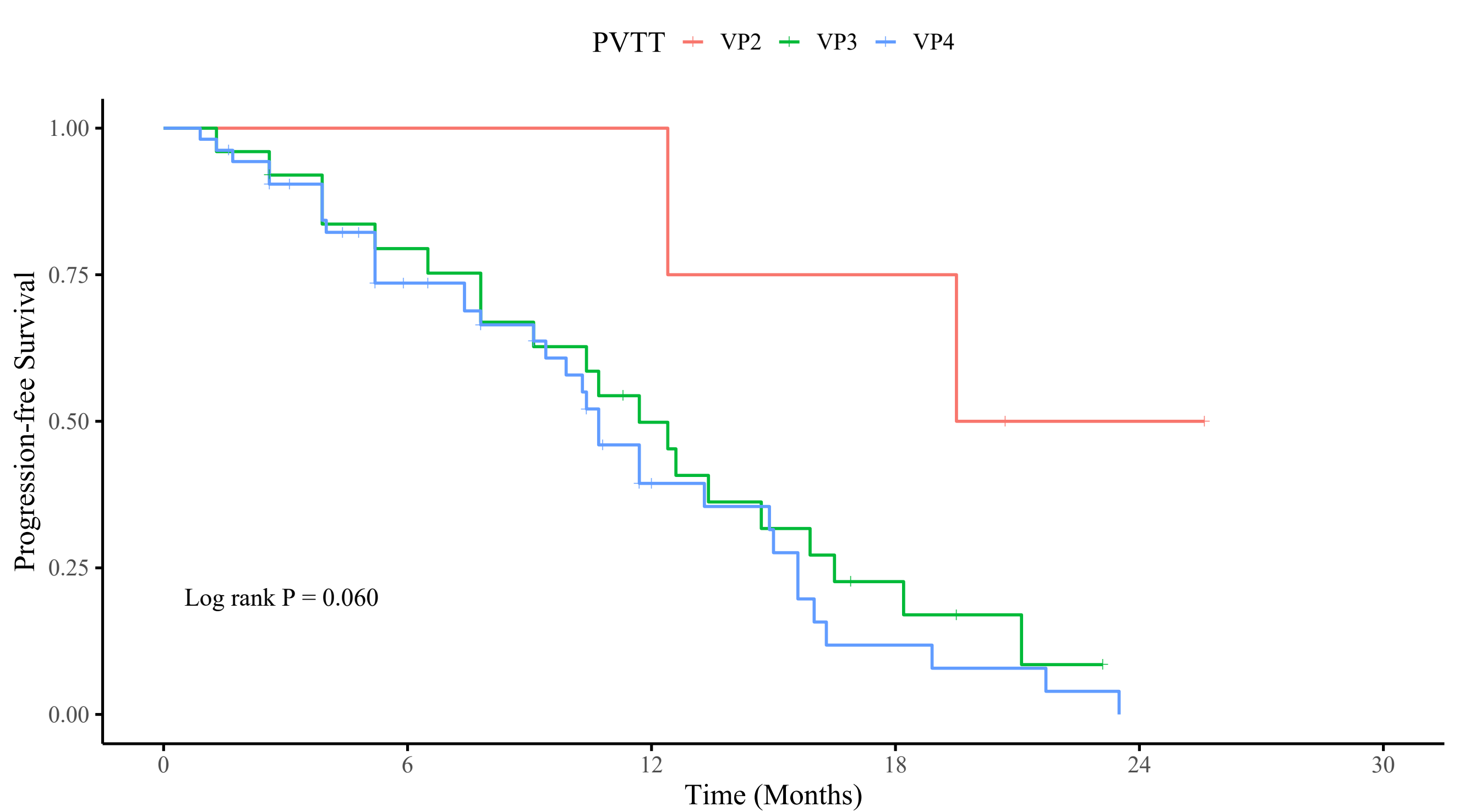

Number at risk

|     |    |    |    |   |   |   |
|-----|----|----|----|---|---|---|
| VP2 | 4  | 4  | 4  | 3 | 1 | 0 |
| VP3 | 25 | 19 | 11 | 4 | 0 | 0 |
| VP4 | 53 | 32 | 11 | 3 | 0 | 0 |

Supplement: Supplementary file 1 [file biomedicines-12-02124-s001.zip › Supplementary Figure S2B.pdf]

PVTT + VP2 + VP3 + VP4

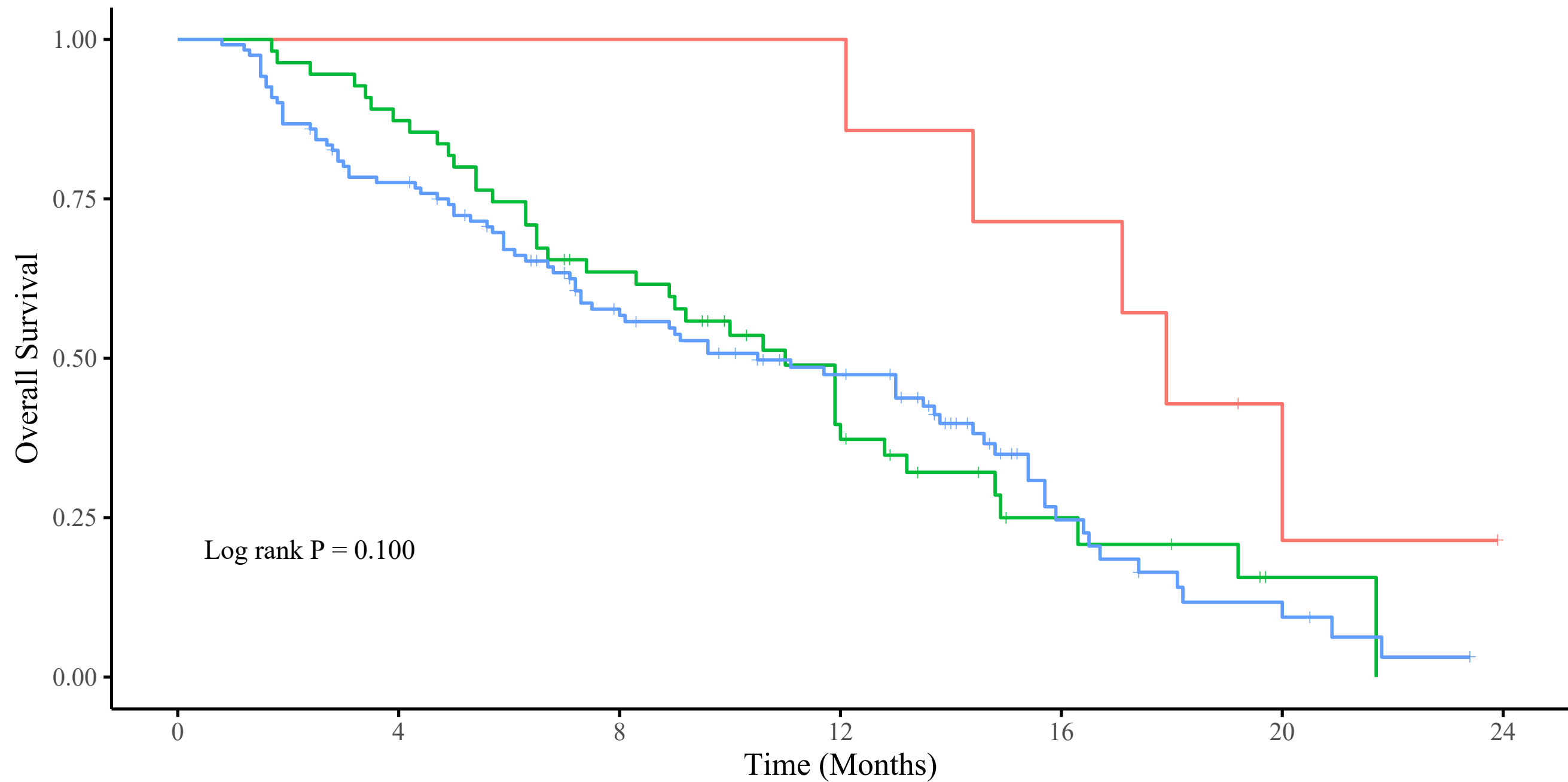

Number at risk

|     |     |    |    |    |    |   |   |
|-----|-----|----|----|----|----|---|---|
| VP2 | 7   | 7  | 7  | 7  | 5  | 2 | 0 |
| VP3 | 55  | 48 | 33 | 17 | 6  | 1 | 0 |
| VP4 | 121 | 92 | 59 | 41 | 12 | 5 | 0 |

Supplement: Supplementary file 1 [file biomedicines-12-02124-s001.zip › Supplementary Figure S3A.pdf]

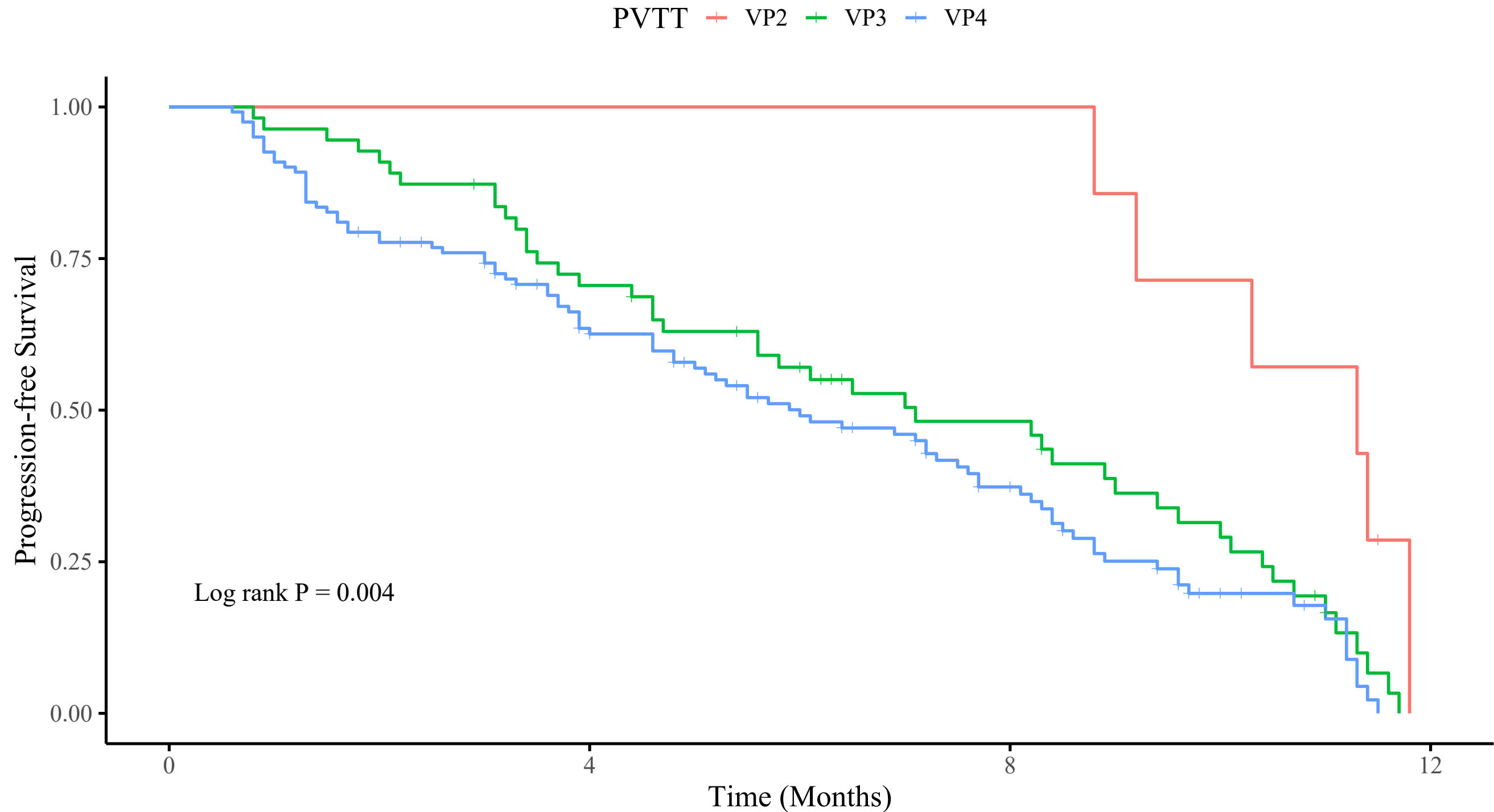

### Number at risk

|     |     |    |    |   |
|-----|-----|----|----|---|
| VP2 | 7   | 7  | 7  | 0 |
| VP3 | 55  | 38 | 21 | 0 |
| VP4 | 121 | 69 | 33 | 0 |

Supplement: Supplementary file 1 [file biomedicines-12-02124-s001.zip › Supplementary Figure S3B.pdf]
